# Thermofield Effects in Graphite-like Amorphous Carbon Films with Nanoscale Structure

**DOI:** 10.3390/ma19101965

**Published:** 2026-05-10

**Authors:** Ekaterina N. Muratova, Igor A. Vrublevsky, Vyacheslav A. Moshnikov, Dmitry A. Kozodaev, Alena Yu. Gagarina, Stepan E. Parfenovich, Danila A. Kavalenka

**Affiliations:** 1 Microelectronics Department, Saint Petersburg Electrotechnical University “LETI”, Professora Popova St., 5, 197022 Saint Petersburg, Russia; vamoshnikov@mail.ru (V.A.M.); gagarina.au@gmail.com (A.Y.G.); stepan.parfenovich@gmail.com (S.E.P.); 2 Micro and Nanoelectronics Department, Belarusian State University of Informatics and Radioelectronics, Brovki St., 6, 220013 Minsk, Belarus; vrublevsky@bsuir.edu.by (I.A.V.); kavalenka.d.a@gmail.com (D.A.K.); 3 NT-MDT BV, Hoenderparkweg 96 b, 7335 Apeldoorn, The Netherlands; kozodaev@ntmdt.nl

**Keywords:** graphite-like carbon films, amorphous carbon, graphite nanoclusters, current–voltage characteristics, space-charge-limited current, Schottky barrier, tunneling, thermionic emission, Poole–Frenkel effect, hopping conductivity mechanism

## Abstract

The paper presents the results of a study on the structure and electrical properties of graphite-like amorphous carbon films deposited by electron-beam evaporation with vacuum heat treatment. The current–voltage characteristics of the films were analyzed in weak and strong electric fields in the temperature range from 25 to 155 °C. For the contact of carbon films with nickel, the Schottky barrier height was calculated based on the obtained current–voltage characteristics. It was found that in the temperature range of 25–45 °C, the mechanism of direct tunneling of charge carriers through the narrow Schottky barrier dominates (*φ_b_* = 0.055 eV). In the range of 55–75 °C, a transition to the thermally assisted tunneling mechanism is observed (*φ_b_* = 0.076 eV). At temperatures above 85 °C, charge carrier transport through the Schottky barrier occurs via thermionic emission (*φ_b_* = 0.3 eV). The analysis of the current–voltage characteristics of graphite-like carbon films allowed us to establish the main mechanisms of hopping conductivity via localized states. It is shown that in the temperature range of 298–348 K, conductivity is determined by states near the Fermi level. The temperature interval of 348–428 K corresponds to conductivity through the band tail of localized states near the conduction band. It is shown that the increase in conductivity in strong electric fields is due to the Poole–Frenkel effect.

## 1. Introduction

In recent decades, thin carbon films have attracted significant interest from researchers due to the unique combination of their structural and physical properties [1,2,3,4,5]. Among the main advantages of amorphous carbon films are their single-component composition and the possibility of their formation using vacuum deposition methods, resulting in an amorphous material structure.

According to the literature, such films represent a two-phase system, including a diamond-like component with *sp*^3^-hybridized carbon atoms in tetrahedral coordination, as well as a graphite-like phase with *sp*^2^ hybridization. The graphite-like regions form nanoscale *sp*^2^ clusters, with characteristic sizes ranging from a few nanometers [6,7].

In graphite-like amorphous carbon films, *sp*^2^-hybridized carbon atoms predominate, with the *sp*^2^/*sp*^3^ ratio determining the material’s properties. The structure of such films consists of disordered graphite nanoclusters that lack the long-range order characteristic of crystalline graphite but retain its local properties. Due to the high proportion of *sp*^2^-bonds, these films exhibit relatively high electrical conductivity. The synthesis of graphite-like amorphous carbon is typically carried out using methods such as electron-beam evaporation, magnetron sputtering, or pulsed laser deposition.

The electrical properties of such films depend on the ratio of *sp*^2^- and *sp*^3^-hybridized carbon atoms and can vary widely—from semimetallic (characteristic of graphite) to dielectric (characteristic of diamond) [8,9,10].

Several theoretical models have been proposed to describe the electrical conductivity mechanisms in amorphous carbon. These include multiphonon tunneling, variable-range hopping conductivity through band tail states, and hopping conductivity via localized states near the Fermi level.

The effect of temperature on the electrical transport properties of amorphous carbon films has been investigated previously [11,12,13,14,15]. It has been shown that the current–voltage characteristics of amorphous carbon films exhibit a complex temperature dependence governed by factors such as the film structure, the ratio of *sp*^2^- and *sp*^3^-hybridized carbon, the dominant conduction mechanisms, and the deposition conditions.

However, the number of studies dedicated to investigating the influence of temperature on the conductivity mechanisms in graphite-like amorphous carbon films remains limited. Furthermore, the specific mechanisms governing charge carrier transport across Schottky barriers formed with amorphous carbon films remain largely unexplored.

As a result of the synergy between conductive properties, low density, and low cost, carbon films are considered a promising material for various technical applications. In particular, they can be used in polymer composites, supercapacitor elements, field emission devices, and equipment designed for operation at elevated temperatures [16,17,18].

The present study is focused on the morphology of graphite-like amorphous carbon films, investigating the influence of temperature on the processes by which charge carriers overcome the Schottky barrier, determining the energy parameters of localized states in the bandgap, and establishing the conductivity mechanisms.

## 2. Materials and Methods

Thin films of graphite-like carbon (*a-C*) with a thickness of 50 nm were deposited onto silicon substrates (111) with a thermal SiO_2_ layer and a 100 nm thick nickel layer by electron-beam evaporation. The deposition conditions are described in detail in our previous paper [19].

The obtained films were subjected to rapid thermal annealing in vacuum at 700 °C for 20 s. After annealing, the electrical resistance of the films decreased by at least a factor of 19. As shown in our paper [19], Raman spectra of such films after heat treatment exhibit splitting with the appearance of peaks, indicating graphitization of the material.

Nickel contacts with an area of 800 × 800 μm and a thickness of 100 nm were formed on the surface of the obtained films.

The C–V characteristics of the samples were measured using a TH512 semiconductor capacitance–voltage analyzer (Tonghui Electronics Co., Ltd., Changzhou, China).

**Figure 1:**
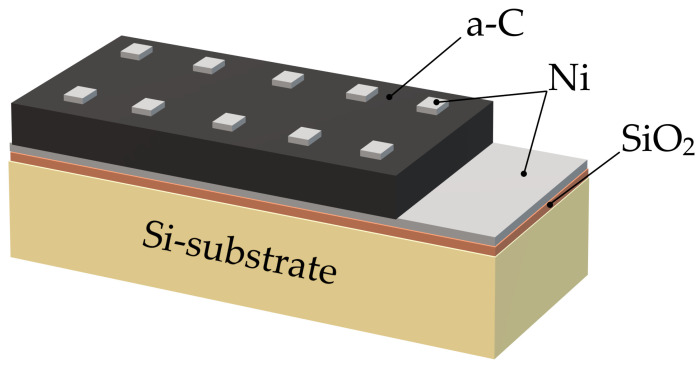
The schematic illustration of the Ni/a-C/Ni Schottky structure.

Figure 1 shows the schematic illustration of the Ni/a-C/Ni structure with nickel (Ni) metal for Schottky contacts.

Surface morphology of the graphite-like carbon films was studied using atomic force microscopy (AFM) in scanning mode with an NTEGRA PRIMA microscope (NT-MDT Europe BV, Apeldoorn, The Netherlands) and an NSG01 probe.

The electrical properties of the obtained films were studied by measuring and analyzing current–voltage (I-V) characteristics using a SEMISHARE MG probe station (SEMISHARE Technology, Changzhou, China) and a Progress-3000 parameter analyzer (NPK Progress, Moscow, Russia).

## 3. Results and Discussion

**Figure 2:**
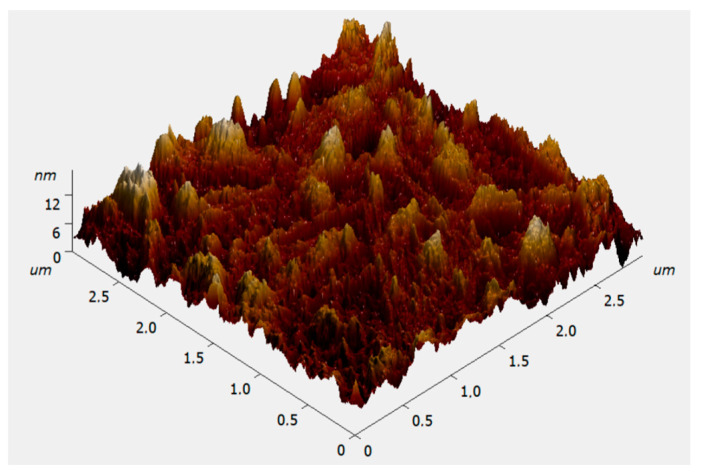
Surface topography of the deposited graphite-like carbon films as revealed by atomic force microscopy.

Figure 2 shows surface topography of the deposited graphite-like carbon films as revealed by atomic force microscopy. Statistical analysis data indicate that over a scanning area of 3 × 3 μm, the root-mean-square surface roughness and grain size are 2.575 nm (not exceeding 2.6 nm).

Assuming that the formation of the graphite-like film occurs through layer-by-layer deposition of *sp*^2^-bonded carbon clusters oriented parallel to the substrate, then the film’s grain size corresponds to the size of such nanoclusters localized at the boundaries of *sp*^3^-bonded regions and is ≈ 2.6 nm.

**Figure 3:**
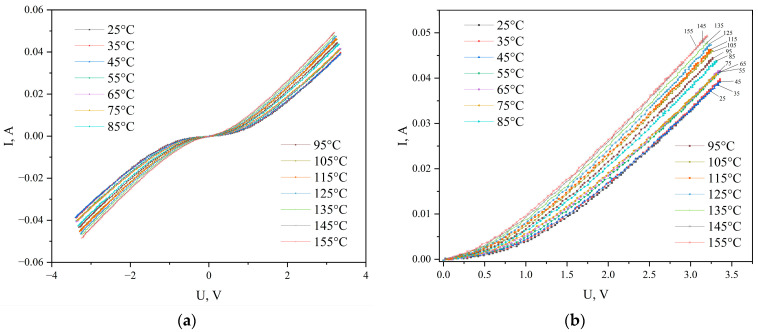
I-V characteristics of the Ni/a-C/Ni structure at: (**a**) negative and positive bias voltage and (**b**) positive bias voltage.

Figure 3a shows the current–voltage (I-V) curves of the Ni/a-C/Ni structure measured at various temperatures in the voltage range from −3.5 to +3.5 V. The I-V curves are symmetric with respect to zero voltage and independent of its polarity over the entire investigated temperature range. Therefore, for further analysis of the charge transport mechanisms, the branch at positive bias voltage was used (Figure 3b).

The analysis of the I-V characteristics vs. temperature, presented in Figure 3b, revealed several distinct characteristic intervals. In the range of 25–45 °C, the current curves show practically no temperature dependence, which, according to the model of tunneling charge carrier transport through the Schottky barrier, indicates the dominance of the tunneling conductivity mechanism [20,21,22]. An increase in temperature to 55 °C is accompanied by an increase in current. Upon further temperature increase from 55 to 75 °C, the current curves again exhibit no temperature dependence, also indicating a tunneling mechanism for charge carrier transport through the Schottky barrier.

Starting from 85 °C, the nature of the I-V characteristics qualitatively changes: the curves for 85, 95, and 105 °C show a strong temperature dependence, typical of space-charge-limited current (SCLC) [23].

Above 105 °C, the I-V curves demonstrate a weak temperature dependence, indicating a change in the conductivity mechanism and a weakening of its connection with electron traps. As shown in [24,25], an increase in temperature leads to a rise in the trap levels with the highest energy toward the conduction band bottom. This can be explained by the fact that electrons trapped in traps may possess sufficient thermal energy to transition from the upper level of the electron traps into the conduction band. Therefore, for temperatures above 105 °C, the I-V curves already show a weak dependence on temperature.

The obtained results suggest that at a temperature of 105 °C and above, electrons gain an energy *kT* equal to 0.033 eV, which is adequate to excite them into the conduction band. Therefore, it can be considered that the energy of the upper level of electron traps in graphite-like carbon is approximately 0.033 eV. For temperatures of 85 °C and 95 °C, the thermal energy *kT* is 0.031 and 0.032 eV, respectively, which, based on the behavior of the I-V characteristics, is inadequate to excite electrons into the conduction band.

To analyze the obtained I-V curves for a metal/n-type semiconductor contact, it is necessary first to consider the onset of the Schottky barrier.

When a metal/n-type semiconductor contact is formed, if the Fermi level of the metal (Ni, work function 5.0–5.2 eV) lies below the Fermi level of the semiconductor (graphite-like amorphous carbon, work function 4.6 eV) [26,27], a directed transfer of electrons from the semiconductor to the metal occurs during their approach. This transfer continues until the Fermi levels align. As a result, a layer depleted of free charge carriers is formed in the near-contact region of the semiconductor. The positive charges of ionized donors created in this layer are localized near crystal lattice defects and cannot move. This layer has high electrical resistance and is called the depletion layer or space charge region.

The generation of a positive space charge in the semiconductor is accompanied by a bending of the energy bands, leading to the creation of a potential barrier at the interface—the Schottky barrier.

When an external voltage is applied to the metal–semiconductor structure, the energy levels of the semiconductor shift, and the Fermi level decreases. An electric current arises in the closed circuit. Since the main voltage drop occurs across the depletion layer, which has the highest resistance, the voltage drop across the remaining parts of the circuit can be neglected.

Temperature significantly influences the current flow through the Schottky barrier, and the nature of this influence differs for various charge transport mechanisms. Three main conductivity mechanisms are distinguished:

1. Direct tunneling. Charge carriers pass through a narrow potential barrier. The tunneling probability depends exponentially on the barrier thickness and is practically independent of temperature, as the process is governed by quantum mechanical factors.
2. Thermally assisted tunneling. Electrons are thermally excited to levels close to the top of the barrier and then tunnel through the remaining narrow part. If the space charge region is so narrow that electron motion is restricted in one direction, size quantization may occur. In such a narrow potential well, the electron energy takes on discrete values, altering the density of states available for tunneling.
3. Over-barrier transport or thermionic emission. In the classical view, an electron must “jump” over the barrier, requiring an activation energy. Due to thermal energy *kT*, electrons in the semiconductor are excited to levels exceeding the Schottky barrier height. In this case, the current density is highly temperature-dependent and is described by the Richardson Equation (1) [23,28]:

(1) $$J=A^{*}T^{2}exp\left(\frac{-q\varphi_{b}}{kT}\right)$$

where *A* is the thermionic constant, *q* is the electron charge, *k* is Boltzmann’s constant, *T* is the temperature, *φ_b_* is the Schottky barrier height, and *U* is the applied bias voltage.

**Figure 4:**
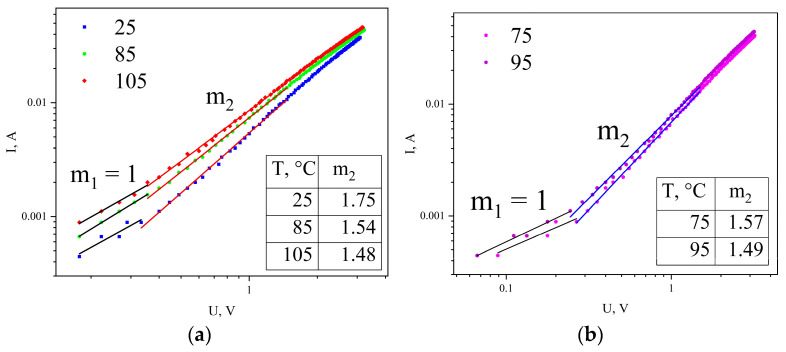
I-V characteristics of the Ni/a-C/Ni structure in double logarithmic coordinates. (**a**) for 25, 85 and 105 °C (**b**) for 75 and 95 °C. Black line - ohmic region, red and blue lines - SCLC regions.

To determine the conductivity mechanism, the I-V characteristics were plotted in double logarithmic coordinates, allowing the identification of linear sections corresponding to a power-law dependence of current on voltage *I ∝ U^m^*. Figure 4 shows the I-V characteristics for temperatures of 25, 75, 85, 95, and 105 °C, at which, as shown above, different charge carrier transport mechanisms through the Schottky barrier are realized.

As seen in Figure 4, regardless of temperature, two characteristic regions can be distinguished on all forward I-V branches. The first region with exponent *m*_1_ = 1 corresponds to the low electric field region, where conductivity obeys Ohm’s law and is associated with carrier transport through delocalized states. The second region with *m*_2_ = 1.48–1.75 corresponds to the medium field region, where conductivity is determined by injection currents limited by space charge (space-charge-limited current, SCLC).

For the tunneling transport mechanism through the Schottky barrier, the exponent in the second region is *m*_2_ = 1.75 (*T* = 25 °C) and *m*_2_ = 1.67 (*T* = 55 °C). For the thermionic emission mechanism (*T* = 75, 85, 95, 105 °C), the value of *m*_2_ is close to 1.5 or 3/2, which corresponds to the classical “three-halves power law” for current in diodes operating in the space-charge-limited regime.

According to this law, electrons emitted from the cathode do not immediately reach the anode but form a region of negative space charge near the cathode. This charge repels new electrons attempting to leave the cathode, and as a result, the emission current density becomes proportional to U32 [29].

Similar behavior is observed for graphite-like carbon films in the over-barrier emission regime for sample temperatures *T* = 75, 85, 95, 105 °C. This emission current behavior can be explained by the formation of space charge within the film volume, which, due to carrier capture at localized states (traps), limits further emission from the electrode, similar to the diode case. As is known, in the bandgap of semiconductors and dielectrics, localized states arise due to crystal lattice defects or a disordered structure (e.g., in amorphous materials). An electron captured at such a level remains localized until it receives sufficient energy to transition back to the conduction band.

In semiconductors under strong electric fields, the Poole–Frenkel effect occurs [25]. This effect results in a lowering of the activation energy of localized levels by the electric field, leading to an increase in the charge carrier concentration in semiconductors.

Let us consider the Poole–Frenkel effect (field-enhanced thermionic emission) in graphite-like carbon films. The influence of temperature on Poole–Frenkel currents occurs in that an increase in thermal energy contributes to an increase in the number of electrons capable of transitioning from trap levels to the conduction band. As temperature rises, both the probability of electrons overcoming the barrier and the current amplitude increase.

**Figure 5:**
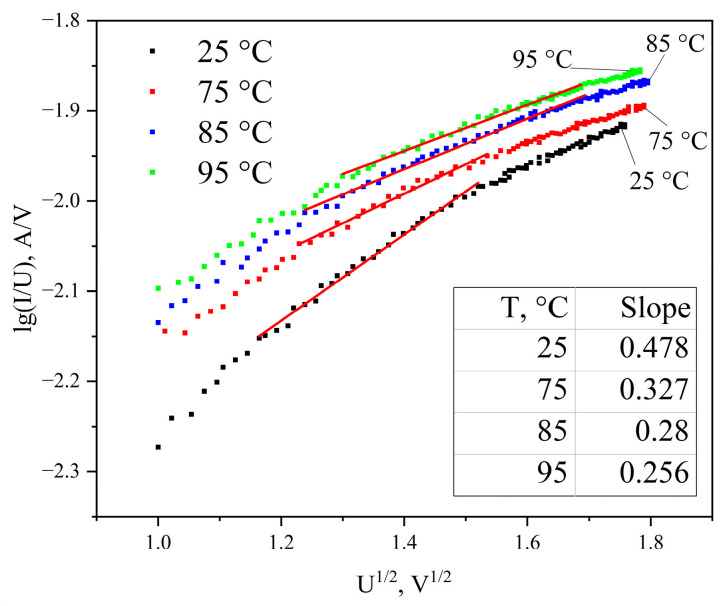
Current–voltage characteristics of the Ni/a-C/Ni structure in Poole–Frenkel coordinates (approximation of experimental points in the bias voltage range from 1.4 to 2.9 V). Red line - Pool-Frenkel current region.

Figure 5 shows the sections of the I-V characteristics corresponding to the Poole–Frenkel mechanism for sample temperatures of 25, 75, 85, and 95 °C.

Analysis of the obtained data shows that at electric field strengths above 2.8·10^5^ V/cm (bias voltage above 1.4 V), the Poole–Frenkel effect occurs. As temperature decreases, the slope of the lines *lg* IU = *f* (U12) increases.

**Figure 6:**
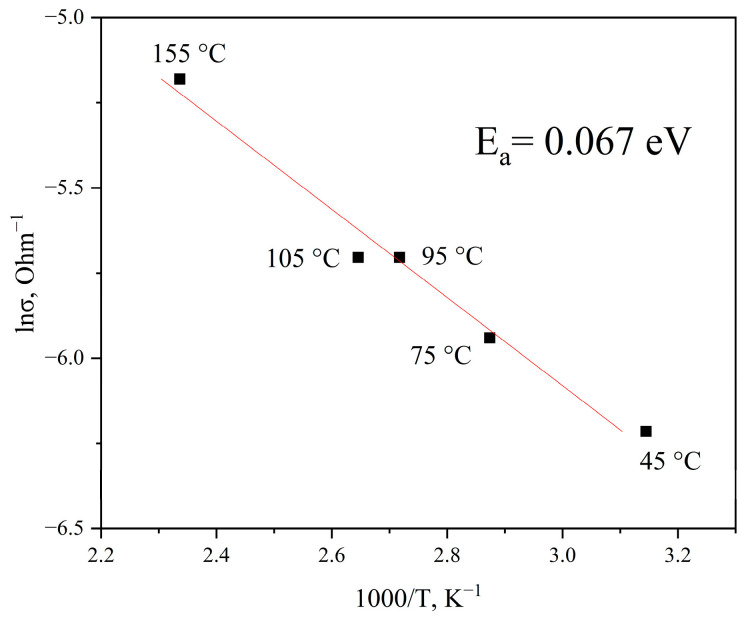
Arrhenius plot for the temperature dependence of conductivity (ohmic region) for graphite-like carbon films.

The conductivity values obtained from the ohmic regions of the I-V characteristics were used to plot the curve of the logarithm of conductivity (*ln σ*) on inverse temperature (1000/*T*), shown in Figure 6.

The slope of the resulting curve corresponds to the activation energy for conductivity via delocalized states, which was found to be *E_a_* = 0.067 eV. This value is in good agreement with data from [30], where an activation energy of *E_a_* = 0.07 eV was obtained for amorphous carbon films after thermal treatment at 700–750 °C.

Let us consider how, using the basic principles of thermionic emission theory and the example of an n-type semiconductor, the Schottky barrier height can be determined.

According to the thermionic emission theory, the current density for an applied voltage *V* is given by [28]

(2) $$J=J_{s}(e^{\frac{qU}{kT}}-1)$$

Here, *J_s_* is the saturation current, *U* is the applied voltage, and *T* is the absolute temperature. Where

(3) $$J_{s}=AT^{2}\left(-e^{\frac{\varphi_{b}}{kT}}\right)$$

Here, *A* is the Richardson constant. At forward bias values exceeding *3kT/q*, the reverse current component becomes insignificant. Consequently, the expression for the current density can be approximated as:

(4) $$J=J_{s}(e^{\frac{qU}{kT}})$$

**Figure 7:**
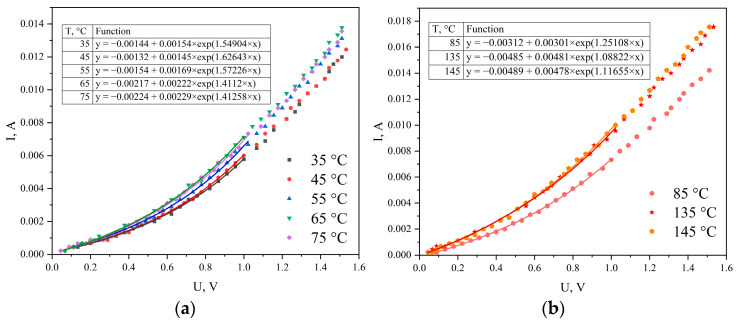
Approximation of I-V curves of the Ni/a-C/Ni structure with an exponential function. (**a**) for 35,45,55,65 and 75 °C(**b**) for 85, 135 and 145 °C.

Approximation of the I-V characteristics for temperatures 35, 45, 55, 65, 75 °C and for 85, 135, 145 °C yields the expressions shown in Figure 7a and Figure 7b, respectively.

Then, using two equations for two nearest temperature values, the Schottky barrier height can be determined. For the temperature pairs 35 and 45 °C, 45 and 55 °C, the value *φ_b_* = 0.055 eV was obtained; for the pairs 55 and 65 °C, 65 and 75 °C—0.076 and 0.078 eV, respectively. For the pair 75 and 85 °C, the barrier height was 0.27 eV, and for the pair 135 and 145 °C—0.308 eV.

The results of the analysis of the temperature influence on the current curves and Schottky barrier height are summarized in Table 1.

As seen from the table, at a Schottky barrier height of 0.055 eV, the tunneling mechanism dominates the current flow through the contact. An increase in temperature to 55 °C and the associated energy quantization lead to an increase in its height to 0.076–0.078 eV, which also corresponds to the tunneling mechanism under conditions of size quantization. A further increase in sample temperature is accompanied by a transition to thermionic emission, as evidenced by the increase in barrier height to 0.27–0.308 eV and the strong temperature dependence of the current. The low Schottky barrier height in this case indicates a narrowing of the effective band gap in amorphous carbon due to the high content of graphite-like *sp*^2^-bonds.

It should be noted that in the ideal case, a Schottky barrier is characterized by a uniform height and a homogeneous thickness across the entire contact area. However, the experimentally observed scatter in the barrier height values indicates the presence of lateral inhomogeneity of the barrier at various points across the contact interface [31,32].

In the case of a rectifying metal–semiconductor contact, a depletion layer (a region depleted of majority charge carriers) forms in the near-surface region of the semiconductor, creating a potential barrier at the boundary with the metal. For a quantitative analysis of the inhomogeneity of this barrier, it is first necessary to estimate the thickness of the depletion layer, which, like the barrier capacitance, can be determined from capacitance–voltage measurements.

**Figure 8:**
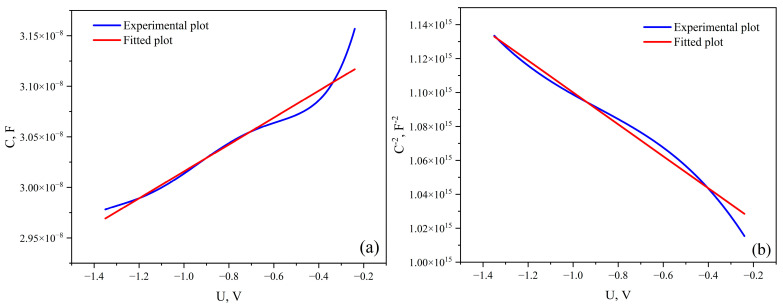
C-V (**a**) and C^−2^-V (**b**) characteristics at 25 °C for Ni/a-C structure.

Figure 8 shows the capacitance *C = f(U)* and *1*/*C*^2^ characteristics as functions of the bias voltage. As can be seen from the curves, an increase in the negative bias leads to a decrease in capacitance and a corresponding increase in *1/C*^2^. This behavior is characteristic of an *n*-type semiconductor. In this case, the application of a negative bias voltage causes the drift of electrons deeper into the material (expansion of the depletion region) and, consequently, a decrease in the differential capacitance. Therefore, the obtained results confirm the *n*-type conductivity of the graphite-like carbon film.

The concentration of immobile charges *N* in the depletion region can be calculated from the slope (∣*tanα*∣) of the linear section of the *1/C^2^ = f(U)* plot according to the expression:

(5) $$N=\frac{2}{\varepsilon \varepsilon_{0}S^{2}|tg\;\alpha |}$$

where *ε*—dielectric constant of niobium pentoxide (Nb_2_O_5_), *ε_0_*—vacuum permittivity (dielectric constant of free space), *S*—contact area (cm^2^), *tanα*—absolute value of the slope of the *1/C^2^ =f(U)* curve.

Assuming a dielectric constant of *ε* = 32 [4] and an experimentally determined value of *tanα*= 5.82 × 10^13^, the calculated concentration of positive immobile charges is *N* = 1.85 × 10^21^ cm^−3^. This value correlates well with literature data [3] on the density of unpaired electron charges in amorphous carbon (*a-C*) obtained by electron spin resonance (ESR), which are on the order of 10^20^ cm^−3^. This indicates that the emergence of a positive space charge region in the depletion layer is due to intrinsic structural defects in the amorphous carbon.

The width of the space charge region *W* can be estimated using the parallel-plate capacitor approximation:

(6) $$W\;=\;\frac{\varepsilon \varepsilon_{0}S}{C}$$

Calculations yield a value of *W* ≈ 6.25 nm, indicating that the actual width of the barrier at the metal interface cannot exceed this value and is confined to a range of several nanometers. At the same time, the experimentally measured root-mean-square surface roughness of the carbon film is 2.6 nm. The comparable scales of the barrier thickness and the surface roughness fluctuations give rise to pronounced local distortions of the electric field at the nanoscale level. Such barrier inhomogeneities create conditions that enhance the probability of charge carrier tunneling through the potential barrier.

Consequently, at room temperature, the underestimated values of the effective Schottky barrier height are due to the contribution of direct tunneling. As the temperature increases to 55 °C, thermal energy begins to assist charge carrier tunneling (thermally assisted tunneling); however, the effective barrier height still retains underestimated values due to the persistence of lateral inhomogeneity. At temperatures above 85 °C, the dominant charge transport mechanism becomes classical thermionic emission, since the thermal energy kT becomes comparable to the potential barrier height, and the contribution of the tunneling current component is effectively suppressed.

**Figure 9:**
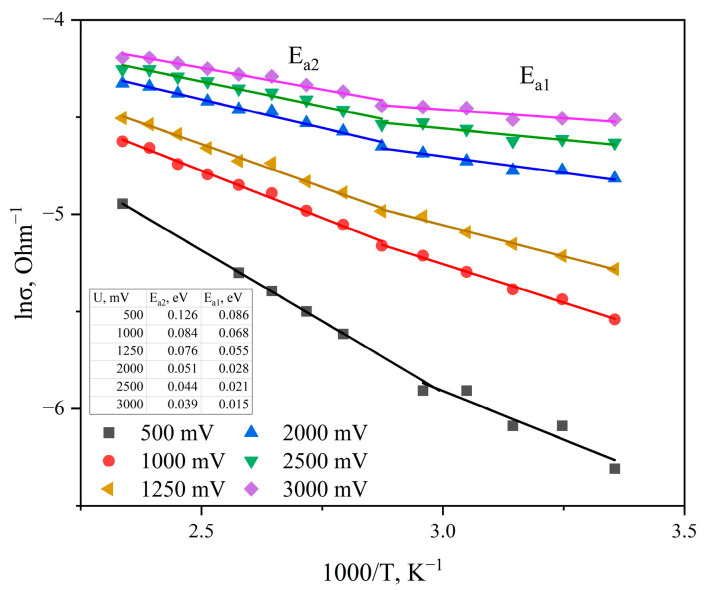
Change in conductivity logarithm ln σ versus inverse temperature 1000/T for various bias voltages for graphite-like carbon films.

Figure 9 shows the temperature dependences of the sample conductivity, plotted in Arrhenius coordinates (ln *σ* = *f* (1000T)) at various bias voltages. The curves were obtained for both the ohmic region of the I-V characteristic and the region of electric fields where the Poole–Frenkel effect is observed.

As can be seen, the sample conductivity exhibits activated behavior over the entire investigated voltage range. Two characteristic regions can be distinguished on all curves: the first region in the temperature range 348–298 K and the second region in the range 428–348 K.

**Figure 10:**
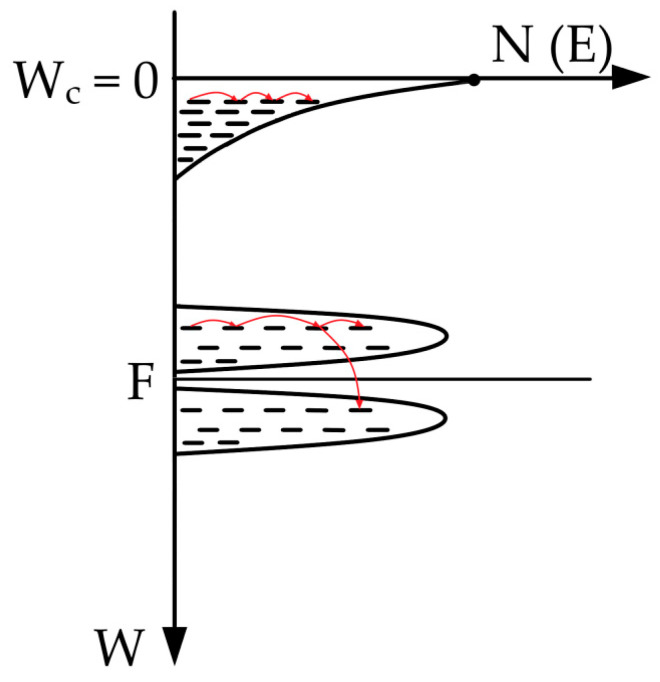
Energy diagram of graphite-like carbon. Red arrows show possible electron jumps.

The obtained dependences can be interpreted within the framework of the band structure model for amorphous materials. As in crystalline semiconductors, amorphous materials possess a valence band *E_v_* and a conduction band *E_c_*, separated by a bandgap. However, due to structural disorder, localized states arise within the bandgap, located near the Fermi level, as well as in the band tails of the density of states at the band edges (Figure 10).

According to this model, three conductivity mechanisms can be distinguished, differing in activation energy:

- Conductivity via delocalized states in the allowed bands;
- Conductivity via localized states in the band tails;
- Hopping conductivity via localized states near the Fermi level.

In the region of localized states, charge carrier transport can occur via hopping of charge carriers between states with different energies. It should also be noted that the mobility of charge carriers in localized states is significantly lower than that in delocalized states. Furthermore, the density of states in the tail is lower than in the allowed band. As a result, activating carriers into the band tail density of states near the conduction band bottom requires significantly higher energy than for hopping conductivity near the Fermi level. Therefore, the first region, starting from room temperature (298–348K), corresponds to hopping conductivity via states near the Fermi level. The second region at higher temperatures (348–428K) is associated with conductivity via the band tail of localized states near the conduction band bottom.

Accordingly, the activation energy *E_a2_*, obtained from the slope of the high-temperature region, relates to conductivity via the band tail, while *E_a1_* relates to hopping conductivity near the Fermi level.

As seen in Figure 9, with increasing bias voltage or increasing electric field strength, the slope of both the high-temperature and low-temperature regions decreases. This indicates a decrease in the effective activation energy of charge carriers under the influence of the electric field.

In amorphous carbon (*a-C*), hopping conductivity and the position of the Fermi level are closely related to the material structure and the *sp*^2^/*sp*^3^ hybridization ratio. In graphite-like amorphous carbon, the primary mechanism at temperatures up to 500 K is variable-range hopping conductivity [26,33]. It is described by Mott’s law, according to which the logarithm of conductivity is linearly dependent on *T*^−1/4^.

**Figure 11:**
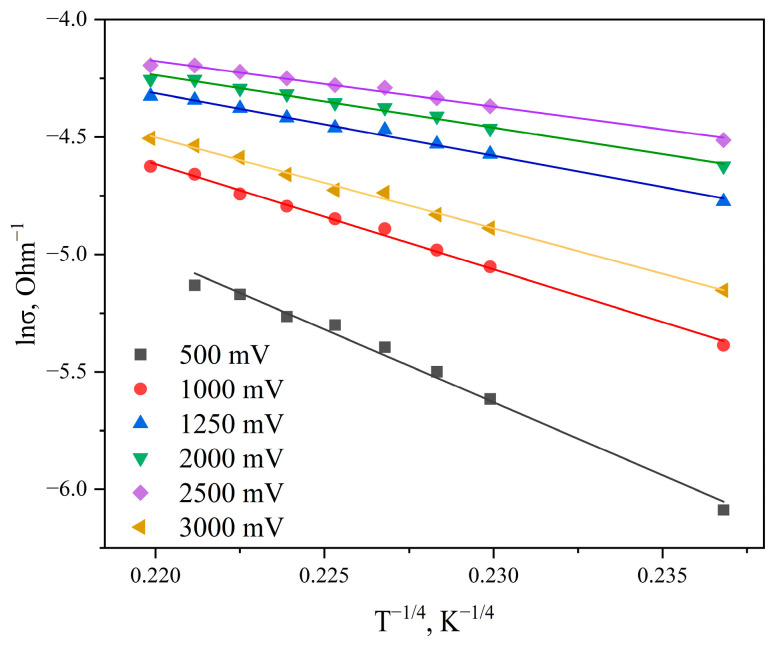
Curves of conductivity change with temperature (*T*^−1/4^) for graphite-like carbon films for applied bias voltages from 500 to 3000 mV.

Figure 11 shows the conductivity curves of the sample plotted in coordinates *lnσ* versus *T*^−1/4^ for various bias voltages.

As seen in Figure 11, the obtained graphs in coordinates ln σ versus *T*^−1/4^ are well approximated by straight lines. This indicates that for all investigated bias voltages (applied electric field strengths), the hopping conductivity mechanism with variable hopping length via localized states is operative.

In graphite-like carbon films, charge transfer occurs via hopping of carriers between *sp*^2^ clusters through dielectric gaps formed by the *sp*^3^-bonded carbon matrix. The external electric field (bias voltage) lowers the energy barrier for hopping in the field direction, leading to a decrease in activation energy with increasing voltage.

**Figure 12:**
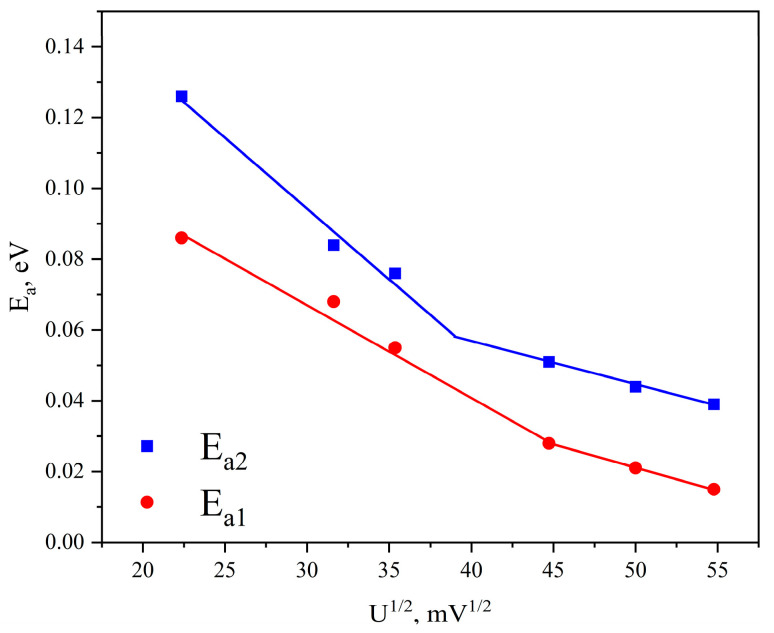
Change in activation energy versus the square root of the bias voltage for graphite-like carbon films.

Figure 12 shows curves of the activation energy for conductivity versus the square root of the applied voltage *U*^1/2^. It is evident that with increasing voltage (electric field), the thermal energy required for charge carriers to overcome the barrier between *sp*^2^ clusters decreases.

Such behavior is characteristic of the hopping conductivity mechanism via localized states. As follows from Figure 12, this mechanism is realized both for conductivity through the band tail of localized states and for conductivity through states near the Fermi level.

## 4. Conclusions

For the contact of carbon films with nickel, the Schottky barrier height was calculated based on the obtained I-V characteristics. It was established that in the temperature range of 25–45 °C, the mechanism of direct tunneling of charge carriers through the narrow Schottky barrier dominates (*φ_b_* = 0.055 eV). In the interval of 55–75 °C, a transition to the thermally assisted tunneling mechanism is observed (*φ_b_* = 0.076 eV). At temperatures above 85 °C, charge carrier transport across the Schottky barrier occurs via thermionic emission (*φ_b_* = 0.3 eV).

Analysis of the current–voltage characteristics of graphite-like carbon films allowed the identification of the main hopping conductivity mechanisms via localized states. It is shown that in the temperature range of 298–348 K, conductivity is determined by states near the Fermi level. The temperature interval in the range of 348–428 K corresponds to conductivity via the band tail of localized states near the conduction band. The obtained results indicate that in graphite-like amorphous carbon at temperatures up to 428 K, the main mechanism is variable-range hopping conductivity. The experimental conductivity curves are in good agreement with Mott’s law, according to which the logarithm of conductivity is linearly dependent on *T*^−1^/^4^.

It was shown that the increase in conductivity in strong electric fields is due to the Poole–Frenkel effect. This conclusion is confirmed by the linear decrease in the activation energy for conductivity with increasing square root of the bias voltage (electric field strength).

## Figures and Tables

**Table 1:** Influence of temperature on current curves and potential barrier height for injection currents in graphite-like amorphous carbon films (a-C) for a contact with Ni.

Contact Materials	Temperature, °C	Slope (m) of Current Line lgI vs lgU	Barrier Height, eV	Influence of Temperature on Current Curve Behavior	Current Flow Mechanism Through Contact
a-C/Ni	25	1.75	-	Independent	Tunneling
	35	1.76	0.055		
	45	-	0.055		
	**Barrier Height Jump**				
	55	1.67	0.076	Independent	Tunneling
	65	-	0.076		
	75	-	0.078		
	**Barrier Height Jump, Change in Temperature Influence on Current Curves**				
	85	1.54 (~3/2)	0.27	Increases	Thermionic Emission
	95	1.49 (~3/2)	-		
	**Change in Temperature Influence on Current Curves**				
	105	-	-	Weakly dependent	Thermionic Emission
	115	-	-		
	125	-	-		
	135	-	0.308		
	145	1.47 (~3/2)	0.308		
	155	-	-		

## Data Availability

The original contributions presented in this study are included in the article. Further inquiries can be directed to the corresponding author.

## Abbreviations

The following abbreviations are used in this manuscript:

AFM	Atomic Force Microscopy
I-V	current–voltage characteristics
SCLC	space-charge-limited current
a-C	amorphous carbon films
